# Electric-field-induced strong enhancement of electroluminescence in multilayer molybdenum disulfide

**DOI:** 10.1038/ncomms8509

**Published:** 2015-07-01

**Authors:** Dehui Li, Rui Cheng, Hailong Zhou, Chen Wang, Anxiang Yin, Yu Chen, Nathan O. Weiss, Yu Huang, Xiangfeng Duan

**Affiliations:** 1 Department of Chemistry and Biochemistry, University of California, Los Angeles, California 90095, USA; 2 Department of Materials Science and Engineering, University of California, Los Angeles, California 90095, USA; 3 California Nanosystems Institute, University of California, Los Angeles, California 90095, USA

## Abstract

The layered transition metal dichalcogenides have attracted considerable interest for their unique electronic and optical properties. While the monolayer MoS_2_ exhibits a direct bandgap, the multilayer MoS_2_ is an indirect bandgap semiconductor and generally optically inactive. Here we report electric-field-induced strong electroluminescence in multilayer MoS_2_. We show that GaN–Al_2_O_3_–MoS_2_ and GaN–Al_2_O_3_–MoS_2_–Al_2_O_3_-graphene vertical heterojunctions can be created with excellent rectification behaviour. Electroluminescence studies demonstrate prominent direct bandgap excitonic emission in multilayer MoS_2_ over the entire vertical junction area. Importantly, the electroluminescence efficiency observed in multilayer MoS_2_ is comparable to or higher than that in monolayers. This strong electroluminescence can be attributed to electric-field-induced carrier redistribution from the lowest energy points (indirect bandgap) to higher energy points (direct bandgap) in *k*-space. The electric-field-induced electroluminescence is general for other layered materials including WSe_2_ and can open up a new pathway towards transition metal dichalcogenide-based optoelectronic devices.

## Abstract (web summary)

The layer-number-dependent optical and electronic properties of transition metal dichalcogenides are promising in optoelectronics. Here, Li *et al.* demonstrate greatly enhanced electroluminescence emission in multi-layer MoS_2_ attributed to the electric-field-induced carrier redistribution.

## Graphical abstract

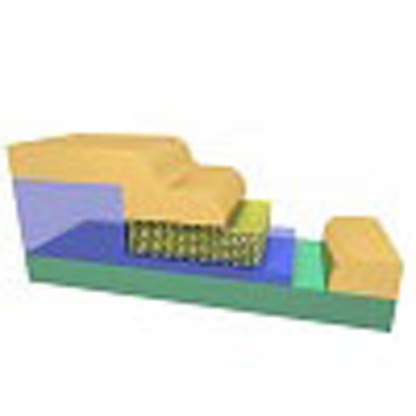

The layered transition metal dichalcogenides (TMDs) have attracted considerable interest for their unique layer-number-dependent electronic and optical properties^1^,^2^,^3^,^4^,^5^,^6^,^7^,^8^,^9^,^10^,^11^,^12^,^13^,^14^,^15^. The monolayer MoS_2_ and WSe_2_ exhibit a direct bandgap with strong photoluminescence and are of particular interest for new types of optoelectronic devices^2^,^9^,^10^,^12^,^16^,^17^,^18^,^19^,^20^. However, the multilayer MoS_2_ is an indirect bandgap semiconductor and is generally believed to be optically inactive, with the photoluminescence typically >3 orders of magnitude weaker than that of the monolayers^2^,^7^, which prevents the multilayer MoS_2_ from being used in the field of light-emitting devices.

To achieve efficient electroluminescence (EL), a p–n junction is usually required to simultaneously inject electrons and holes that recombine to give out photon emission. In case of two-dimensional (2D) TMDs, it is possible to create either a lateral junction with a one-dimensional junction interface or a vertically stacked structure with a 2D junction interface for electron or hole injection, which are necessary for EL emission. The EL emission from the lateral junctions of mono-/few-layer TMDs has been recently reported and is typically limited to the local area across the one-dimensional junction interface^21^,^22^,^23^,^24^,^25^,^26^,^27^, because the full depletion of the monolayer prevents efficient carrier injection throughout the entire monolayer. To achieve broad-area EL emission, the vertically stacked structures with 2D junction interfaces are usually preferred^5^. However, with atomically thin thickness, the rapid carrier leakage^28^ across the monolayer TMDs in the vertical junctions could prevent the efficient recombination of the injected carriers and limit the EL efficiency in the vertically stacked electrically driven light-emitting devices. To this end, multilayer TMDs may be beneficial, which, however, are typically indirect bandgap semiconductors and normally optically inactive. Here we report strong EL emission from the entire 2D junction interface in a unique design of vertical heterostructure devices with multilayer MoS_2_ and WSe_2_ (up to ∼130 layers), and demonstrate a strong electric-field-induced enhancement of the EL in indirect bandgap multilayer MoS_2_ and WSe_2_ to achieve comparable or better efficiency than that of direct bandgap monolayer counterparts.

## Results

### Fabrication of GaN–Al_2_O_3_–Q_3_MoS_2_ structures

**Figure 1:**
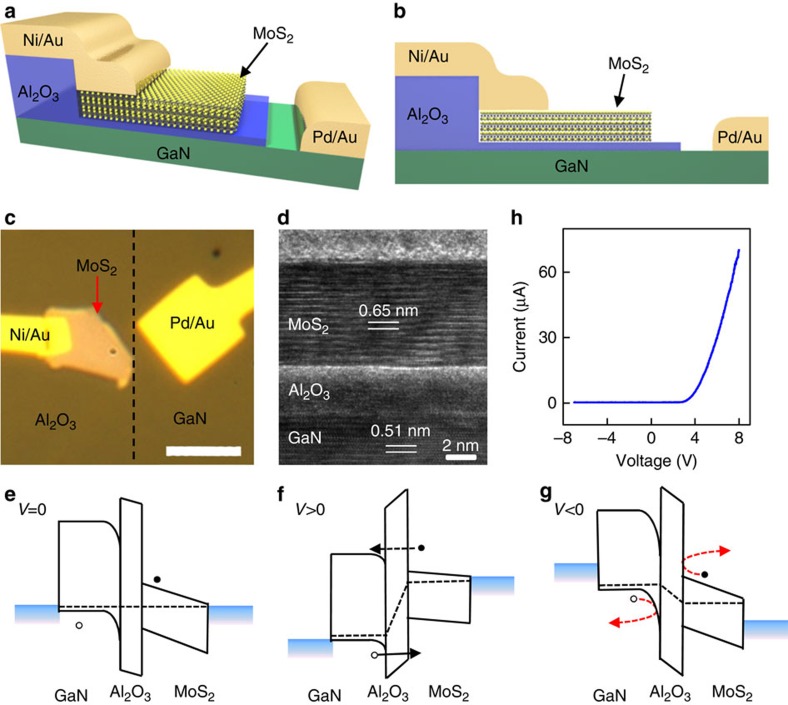
Schematic illustration, structural and electrical characteristics and band diagrams of GaN–Al_2_O_3_–MoS_2_ vertical devices. (**a**) A schematic of the three-dimensional view of the vertically stacked device. (**b**) A schematic of the cross-sectional view of the device. (**c**) An optical image of a GaN–Al_2_O_3_–MoS_2_ vertical device. The dashed line highlights the area with Al_2_O_3_ layer and bare GaN surface. Scale bar, 4 μm. (**d**) A cross-sectional high-resolution transmission electron microscope (TEM) image of the interfaces across the GaN substrate, Al_2_O_3_ and MoS_2_ flake vertical stack. The layer number of the MoS_2_ flake is 14. (**e**) The ideal band diagram of the vertical heterostructure at zero bias. The dashed lines indicate the position of Fermi levels. At zero bias, the bottom of the conduction band and the top of the valence band of MoS_2_ fall within the forbidden bandgap of GaN. (**f**) The ideal band diagram of the vertical heterostructure under forward bias. (**g**) The ideal band diagram of the heterostructure under reverse bias. (**h**) Current versus bias voltage characteristic of a vertically stacked device.

To create vertically stacked light-emitting devices based on n-type MoS_2_, a p-doped GaN is used to inject holes into MoS_2_ flakes (Fig. 1a,b). A thin layer of 4-nm-thick Al_2_O_3_ was deposited on the p-type GaN (∼3.8 μm p-GaN on sapphire) before transferring the mechanically exfoliated MoS_2_ flakes (see Methods). The insulating Al_2_O_3_ layer can partly block the electrons to inject from n-MoS_2_ to p-GaN while allowing holes to be effectively injected from p-GaN to n-MoS_2_ due to the unique band alignment (see band diagram in Fig. 1f,g and further discussion below), where the desired EL occurs^29^. In addition, the insulating layer can also suppress the direct tunnelling of electrons or holes from the top electrode to GaN substrate (through the thin MoS_2_ flakes) to improve the EL efficiency. A Ni/Au metal thin film was used as the top-contact electrodes at the edge of the MoS_2_ and the p-GaN substrate was contacted with a Pd/Au metal thin film (Supplementary Fig. 1). Figure 1c displays an optical image of the top view of a typical device on the GaN substrate, where the MoS_2_ flake and electrodes are indicated. A high-resolution cross-sectional transmission electron microscope image clearly shows the interfaces of the GaN–Al_2_O_3_–MoS_2_ vertical stack (Fig. 1d).

### Output characteristics of GaN–Al_2_O_3_–MoS_2_ structures

Figure 1e–g shows the ideal band diagrams of the vertical heterostructures. At or near the zero bias, the insulating layer blocks both electrons and holes from passing through the heterojunction with a zero current (Fig. 1e). It should be noted that the valence band energy difference between GaN and Al_2_O_3_ (*ΔE*_v_=1 eV) is much smaller than that of the conduction bands (*ΔE*_c_=2.2 eV). As a consequence, it is much easier for holes to be injected from GaN to MoS_2_ flakes due to a relatively lower potential barrier. Applying a positive voltage to p-type GaN will drive the holes to tunnel from GaN to MoS_2_, resulting in a forward current (Fig. 1e). Further increasing the applied positive voltage on the GaN, the holes in GaN can even thermally emit into MoS_2_, with the injection current rapidly increasing with the bias voltage. Under sufficient high voltage, electrons in MoS_2_ are able to tunnel to the conduction band of the GaN, which can also contribute to the forward current and lead to the emission from the GaN substrate. Under a negative bias, the top of the valence band of GaN falls within the bandgap of MoS_2_, while the bottom of the conduction band of MoS_2_ falls within the bandgap of GaN (Fig. 1g). Therefore, there is no available state for both electrons and holes to tunnel through the insulating layer, resulting in zero current. The current–voltage output characteristic (Fig. 1h) shows clear current rectification behaviour, demonstrating excellent diode characteristics in the GaN–Al_2_O_3_–MoS_2_ vertical devices to satisfy the basic requirement for high-efficiency EL devices.

### EL in GaN–Al_2_O_3_–MoS_2_ structures

**Figure 2:**
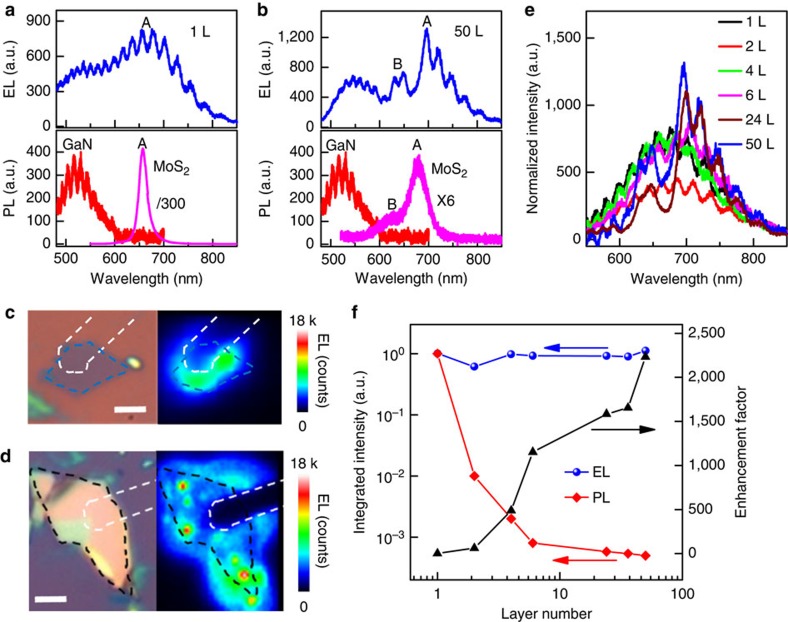
Electroluminescence (EL) from GaN–Al_2_O_3_–MoS_2_ vertical devices. (**a**) The EL spectrum of a monolayer device under an injection current of 30 μA. The PL spectra of the GaN substrate and the same monolayer MoS_2_ flake (divided by 300) are given as well to assign the EL peaks. (**b**) The EL spectrum of a 50-layer MoS_2_ device under an injection current of 88 μA. The PL spectra of the GaN substrate and the same 50-layer MoS_2_ flake (multiplied by 6) are shown as well. The PL intensity of monolayer MoS_2_ is around 2,000 times larger than that of 50-layer MoS_2_. (**c**) The optical image and the corresponding EL mapping for the same monolayer device. The monolayer MoS_2_ flake and electrode are outlined by dashed lines. A 650-nm longpass filter was used for mapping the emission from MoS_2_ only. Scale bar, 3 μm. (**d**) The optical image and the corresponding EL mapping for the same 50-layer MoS_2_ device. The 50-layer MoS_2_ flake and electrode are outlined by dashed lines. Scale bar, 3 μm. A 650-nm longpass filter was used for mapping the emission from MoS_2_ only. (**e**) The EL spectra from MoS_2_ flakes with various number of layers. The spectra have been normalized by the injection current density to compare with each other, and the GaN emission has been subtracted based on a Gaussian fitting. (**f**) The normalized integrated EL and PL intensity (left axis) and the relative enhancement factor defined as the ratio of EL intensity to the PL intensity (right axis) as a function of the layer number.

Importantly, both the monolayer and multilayer MoS_2_ can exhibit clear EL emission under a forward bias. The EL spectra of a monolayer and a 50-layer MoS_2_ device are shown in Fig. 2a,b. To identify and assign the origin of the EL peaks, the photoluminescence (PL) spectra of GaN and MoS_2_ are displayed as well. With close comparison of the EL spectra with the PL spectra, we can unambiguously assign the EL emission peak around 530 nm to the defect emission of the GaN substrate, the peak at 667 nm to the exciton A emission in the monolayer MoS_2_ and the peaks at 691 and 635 nm to the exciton A and B emission, respectively, in the 50-layer MoS_2_ device^30^. The small periodic oscillations can be attributed to the interference patterns formed due to the GaN substrate. For both monolayer and multilayer MoS_2_ devices, the EL peaks show a small redshift compared with the corresponding PL peaks due to a self-heating effect, which is commonly seen in EL spectra of traditional semiconductor heterostructures^31^. It is also noted that the full width at half maximum of EL peaks of MoS_2_ is much broader than that of corresponding PL peaks, which may be attributed to the electric-field-induced peak broadening^32^. To focus on the EL only from MoS_2_, we insert a longpass filter with a cutoff wavelength of 650 nm to eliminate the emission from the GaN substrate. Figure 2c,d shows the optical image and EL mapping for the monolayer and the 50-layer MoS_2_ flakes, respectively. The profile of MoS_2_ flakes and the contact electrodes are outlined by dashed lines to identify the position of the EL. Importantly, bright EL are clearly seen from entire overlapping area of the vertical heterostructures for both the monolayer and multilayer MoS_2_ flakes. The non-uniform distribution of the EL may be attributed to the imperfect interfaces (or impurities) due to the wet transfer process, the surface roughness of the p-GaN substrate and/or non-uniform field distribution.

Next, we have investigated the thickness dependence to probe the influence of thickness on the EL efficiency and understand the mechanism for the enhanced EL in the multilayer indirect bandgap MoS_2_. To properly compare the EL efficiency among different devices, we have normalized the EL spectra by injecting current density, which is proportional to the EL efficiency. Interestingly, the overall normalized intensity for all devices with different MoS_2_ thickness (including the monolayer and thicker one up to 50 layers) is highly comparable to each other (Fig. 2e; Supplementary Fig. 2). Both the exciton A and B can be identified in the EL spectra of MoS_2_ flakes thicker than 6 nm, whereas only exciton A emission can be clearly seen from EL spectra of the thinner flakes, which may be ascribed to the stronger electric-field-induced peak broadening in the thin layers. In the thinner flakes, the electric field is much stronger and the field-induced peak broadening is so severe that the exciton A and B emission merge into one broad peak^32^.

The observation of nearly comparable EL intensity in monolayer and multilayer MoS_2_ is quite striking when considering the PL intensity is strongly dependent on the number of layers in these TMD materials. With a direct bandgap, monolayer can typically exhibit rather strong PL, which deceases rapidly with the increasing number of atomic layers due to the crossover from a direct bandgap semiconductor to an indirect one with increasing layer number^2^,^7^. For the PL process, the photogenerated carriers undergo rapid thermalization and reach thermal equilibrium in a timescale much shorter than that of radiative recombination^33^, with the majority of carriers occupying their immediate band extremum, that is, electrons at *Λ* valley and holes at *Γ* hill of *k*-space in multilayer MoS_2_. As a result, the direct bandgap recombination at the *K* point is rather weak due to the lack of carrier occupation at the *K* point in multilayer MoS_2_ under thermal equilibrium. As the thickness increases, less and less carriers occupy the *K* point due to the increasing energy difference between the *K* valley and *Λ* valley for electrons (*ΔE*_Λ−ϰ_) and between the *K* hill and *Γ* hill for holes (*ΔE*_Γ−ϰ_), resulting in an exponential decrease of the PL intensity with the increase of MoS_2_ thickness^7^.

### Electric-field-induced carrier redistribution

**Figure 3:**
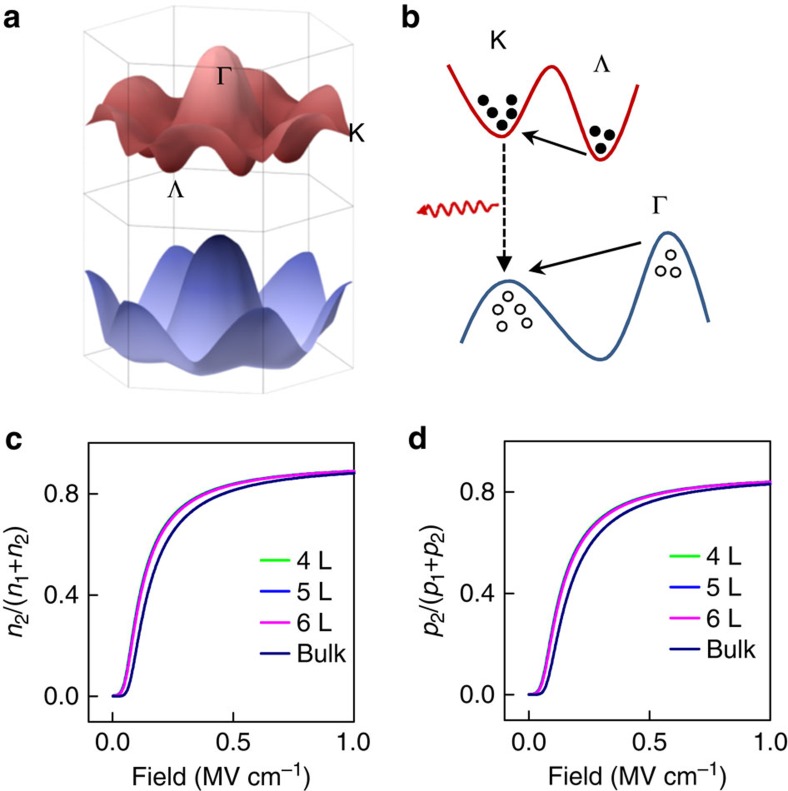
The schematic of the carrier transfer processes and the calculated valley/hill population fraction. (**a**) The schematic illustration of the conduction band and valence band. (**b**) The electric-field-induced carrier transfer between different energy valleys and hills. The equivalent valley number and hill number are 6 at *K* and *Λ* points and 1 at the *Γ* point. (**c**) The calculated electron population fraction at the *K* valley as a function of the applied electric field for different thickness MoS_2_ flakes. (**d**) The calculated hole population fraction at the *K* hill as a function of the applied electric field for different thickness MoS_2_ flakes.

This distinct contrast between EL and PL is particularly evident in the plot of EL and PL versus layer thickness, in which both EL and PL intensity observed in different layered samples are normalized by the EL and PL intensity of the monolayer MoS_2_ (Fig. 2e). If we define the ratio of the EL intensity to PL intensity as a relative electric-field-induced EL enhancement factor, the enhancement factor increases with the increasing MoS_2_ layer number and reaches as large as ∼2,000 for a 50-layer sample (right axis of the Fig. 2f). It should be noted that the EL in all multilayer MoS_2_ originates from the direct bandgap (*ϰ*–*ϰ*) transition rather than the near-band-edge (*Λ*–*Γ*) transition. This can occur due to the fact that the electric field could induce carrier redistribution from low energy points to high energy points of the *k*-space (Fig. 3a,b), leading to a non-equilibrium distribution of the injected carriers in the multilayer indirect bandgap MoS_2_. Under the applied electric field, the injected electrons and holes are accelerated to gain significant kinetic energy. Thus, the electron and hole temperature are significantly higher than that of the environment, causing electrons to transfer from *Λ* valley to *ϰ* valley while holes to transfer from *Γ* hill to *ϰ* hill^24^,^34^. In this way, electrons and holes can recombine at the *K* point, leading to greatly enhanced direct bandgap EL in multilayer MoS_2_.

It should be noted that the indirect bandgap exciton binding energy is rather large for bilayer and trilayer MoS_2_ (around 400 meV for bilayer and around 100 meV for trilayer)^35^, which can greatly suppress the electric-field-induced carrier redistribution. Consequently, EL in bilayer and trilayer MoS_2_ is rather weak. When the thickness is larger than three layers, the indirect bandgap exciton binding energy decreases to about 25 meV^35^. As a result, the indirect bandgap excitons are mostly ionized at room temperature and can be efficiently redistributed to direct bandgap *K* points under high electrical field for EL emission. We have calculated the *K-*point population fraction for electrons *n*_2_/(*n*_1_+*n*_2_) and holes *p*_2_/(*p*_1_+*p*_2_) as a function of the applied electric field for MoS_2_ thicker than 3 layers (Fig. 3c,d; Supplementary Note 1). The *K*-point population fraction for both electrons and holes starts to increase around 40 kV cm^−1^ and saturates at 1 MV cm^−1^ with a saturation value of ∼0.8. Such a large fraction of population at a higher-energy direct bandgap *K* point is quite striking and largely responsible for the enhanced EL emission in multilayer MoS_2_, which is usually difficult to achieve in other indirect bandgap semiconductors (for example, Si), but has been observed in a number of direct bandgap semiconductors (for example, GaAs, InP)^34^,^36^. This unique electric-field-enhanced EL can be attributed to the unique band structure of MoS_2_: the density of states at the *K* point is much larger than that at the *Λ*(*Γ*) point due to the larger effective mass at the *K* valley(hill) and the larger number of equivalent hill numbers at *K* points (6) compared with that at *Γ* points (1)^37^. Indeed, the density-of-state ratio is evaluated to be 6.4 for holes between the *K* hill and *Γ* hill and 10.6 for electrons between *ϰ* valley and *Λ* valley (Supplementary Note 1 and Supplementary Table 1). With much higher density of states at the *K* point in MoS_2_, it is not totally surprising to expect that the majority of the injected carriers can redistribute to the higher-energy *K* valleys or hills, which is responsible for the enhanced EL in our devices.

### GaN–Al_2_O_3_–MoS_2_–Al_2_O_3_-graphene structures

**Figure 4:**
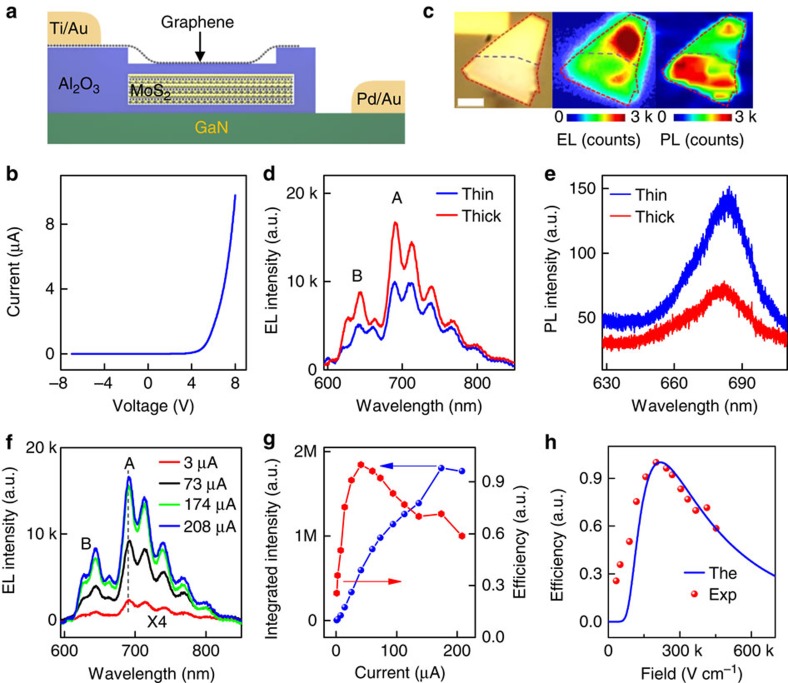
EL from vertically stacked GaN–Al_2_O_3_–MoS_2_–Al_2_O_3_-graphene heterostructures. (**a**) A schematic illustration of the cross-sectional view of the GaN–Al_2_O_3_–MoS_2_–Al_2_O_3_-graphene vertical heterostructure. (**b**) Current versus bias voltage characteristic of the GaN–Al_2_O_3_–MoS_2_–Al_2_O_3_-graphene vertical device. (**c**) The optical image, EL mapping (under an injection current of 8 μA) and PL mapping ( excited by a 514-nm Ar ion laser) of a multilayer device. The MoS_2_ flake is composed of two parts with different thicknesses: the 36-nm-thick lower part and the 92-nm-thick upper part. Scale bar, 3 μm. (**d**) EL spectra from the thick part and thin part of the MoS_2_ flake under an injection current of 174 μA. The GaN emission has been subtracted based on a Gaussian fitting. (**e**) PL spectra from the thick part and thin part of the same MoS_2_ flake. (**f**) EL spectra of the thick part at different injection currents. The GaN emission has been subtracted based on the Gaussian fitting. The corresponding applied voltages are 6, 13, 17 and 18 V. (**g**) The integrated EL intensity and EL efficiency as a function of the injection current for the thick part. (**h**) The EL efficiency as a function of the electric field. The discrete points are experimental results and the solid line is the theoretical calculation.

With the GaN–Al_2_O_3_–MoS_2_ structures, a metal electrode is deposited on the edge of the MoS_2_ flake for electron injection. Due to the resistivity and depletion of MoS_2_ along both the lateral and vertical direction, there are both vertical and lateral components of electric field. It is therefore difficult to precisely determine the exact electrical field and quantitatively correlate the experimental results with the theoretical ones. To further verify the proposed mechanism, we have created vertically stacked GaN–Al_2_O_3_–MoS_2_–Al_2_O_3_-graphene heterostructures (Fig. 4a), in which graphene is used as the top electrode. In this case, the applied electric field is largely dominated by the vertical component. We have also included a 4-nm-thick Al_2_O_3_ layer between the MoS_2_ and graphene in the new heterostructures to prevent electrons and holes from directly transferring to graphene, which would quench the EL in MoS_2_. The current–voltage characteristic of this new structure also shows excellent diode behaviour (Fig. 4b). However, the injection current is typically several times smaller than that of previous GaN–Al_2_O_3_–MoS_2_ structures at the same bias voltage due to the additional tunnelling layer between MoS_2_ and graphene.

### EL in GaN–Al_2_O_3_–MoS_2_–Al_2_O_3_-graphene structures

Figure 4c displays the optical image, EL mapping and PL mapping of a typical device with the MoS_2_ flake composed of two parts with different thicknesses (lower part with a thickness of 36 nm and upper part with a thickness of 92 nm). The EL mapping shows several important features: firstly, EL is observed from the entire heterojunction area, confirming the formation of a broad-area vertical junction; secondly the thicker part of the flake exhibits a stronger EL signal than the thinner area, which is in striking contrast to the PL mapping of the same sample in which the thinner part clearly shows a stronger PL emission. EL spectra further confirm that the EL intensity of the thicker part is around 1.5 times stronger than that of the thinner part under the same injection condition (Fig. 4d), while the corresponding PL intensity in the thicker part is <40% of that in the thinner area (Fig. 4e).

We have also investigated the EL characteristics as a function of injection current or vertical electric field. The EL intensity increases monotonously with the injection current and saturates around the injection current of 174 μA (Fig. 4f,g; Supplementary Fig. 3). It is also noted that the EL emission from GaN continues to increase with the increasing injection current after the MoS_2_ emission reaches saturation (Supplementary Fig. 3), suggesting the EL saturation in MoS_2_ under high injection current (field) is a unique feature of MoS_2_. Overall, the EL efficiency in MoS_2_ increases rapidly first and then decreases with the increasing injection current (Fig. 4g) or the increasing electric field (Fig. 4h). The vertical electric field is determined by the current density and carrier mobility (Supplementary Figure 4 and Supplementary Note 2) using a space-charge-limited current model (Supplementary Figure 5 and Supplementary Note 3).

The trend of EL efficiency with increasing electric field may be attributed to two competing factors under high electric field that may affect the emission efficiency in the opposite way: the increase of the electron and hole population fraction at direct bandgap *K* point and increase of carrier temperature. To account for this trend, we calculated the relative EL efficiency under various electric fields by taking into account both the electric-field-induced carrier redistribution and carrier temperature^38^ (blue curve in Fig. 4h; Supplementary Figure 6 and Supplementary Note 1). In general, the electron, hole temperature and their population in the higher-energy *K* points all increase with the increasing electric field. The increase of the electron and hole population faction at the direct bandgap *K* point would result in a higher EL efficiency, while the increasing average carrier temperature could reduce the EL efficiency. Together, these two competing factors could lead to an increase of EL efficiency with the electric field at lower-field regime (where the rapid increase of carrier population at the *K* point dominates the EL efficiency, see Fig. 3c,d and Fig. 4h), followed by a decrease of EL efficiency at higher electric field (where the carrier population at the *K* point saturates, and the effect of the carrier temperature dominates the EL efficiency, see Fig. 3c,d and Fig. 4h). Importantly, the observed EL efficiency first increases and then decreases with the increasing electric field (red dots in Fig. 4h), which is consistent with the trend predicted by the theoretical calculations (blue line in Fig. 4h), except at low-field (small injection current) regime where the space-charge-limited current model used to calculate the electric field underestimates the field in MoS_2_ (Supplementary Figure 6 and Supplementary Note 3). The decrease in EL efficiency may also be partly attributed to the increased electron injection from MoS_2_ to GaN substrate under high electric field^28^, which is supported by the continued increase of GaN emission after the saturation of emission in MoS_2_ under high electric field (Supplementary Fig. 3). In addition, other factors such as field-induced exciton ionization and exciton–exciton annihilation may also contribute to the decrease in EL efficiency under high injection current (field).

### Thickness dependence of the EL efficiency

**Figure 5:**
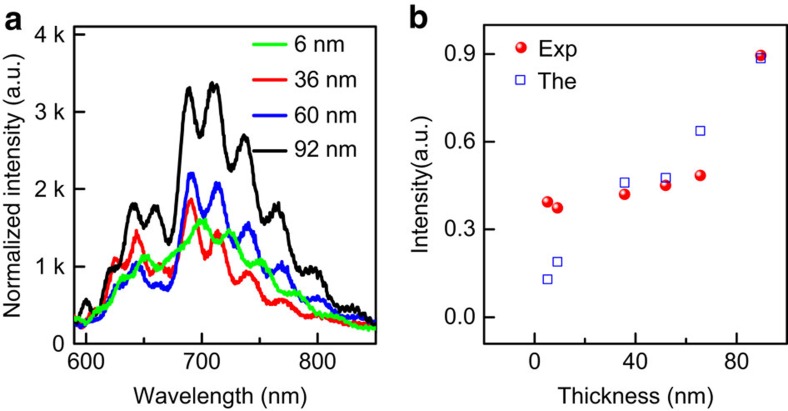
Thickness-dependent EL for vertically stacked GaN–Al_2_O_3_–MoS_2_–Al_2_O_3_-graphene heterostructures. (**a**) The normalized EL spectra for vertical devices with MoS_2_ flakes of varying thickness. The spectra are normalized by the injection of current density to compare with each other. The GaN emission has been subtracted based on a Gaussian fitting. (**b**) The integrated EL intensity as a function of the thickness of the MoS_2_ flakes. The red points are experimental results and the blue hollow squares are obtained from theoretical calculation.

We have further probed the thickness dependence of the EL efficiency based on GaN–Al_2_O_3_–MoS_2_–Al_2_O_3_-graphene heterostructures. The EL intensity normalized by the injection current density indicates that both exciton A and B peaks are present for all flakes with various thicknesses (6–92 nm) (Fig. 5a; Supplementary Fig. 7). The EL efficiency for the current structures is about three times higher than that in the previous GaN–Al_2_O_3_–MoS_2_ structures, likely due to the carrier leakage through the metal electrode in previous structures (Supplementary Fig. 8). Importantly, a clear trend is observed that the normalized integrated EL intensity increases with the increasing flake thickness (Fig. 5b), which might be attributed to at least two factors. First, the applied electric field would be smaller in the thicker flakes for a fixed applied bias voltage, leading to a larger EL efficiency under the same injection current (see Fig. 4g, in the high-field regime where the EL efficiency decreases with increasing electric field). The second factor is the injected carrier leakage^28^. The thicker the flake is, the smaller the percentage of the injected carrier can leak away from the active emitters, resulting in a higher EL efficiency. The theoretical calculation based on the above discussion agrees well with the experimental results (Fig. 5b; Supplementary Note 4). The relatively large discrepancy for the thinner flakes may arise from the inaccurate energy difference *ΔE*_Λ−ϰ_ and *ΔE*_Γ−ϰ_ and effective mass that can be different from the bulk values used in the calculation^39^.

## Discussion

After calibrating the collection efficiency of our EL measurement system, we have estimated the EL external quantum efficiency of our vertical heterostructure devices to be around 10^−4^, which is about one order of magnitude larger than the EL efficiency in monolayer MoS_2_ transistors^26^ and on the same order as the reported value in monolayer WSe_2_ planar structures^23^. We believed this enhanced EL in multilayer indirect bandgap MoS_2_ flakes can be attributed to the unique energy band structure of MoS_2_ and the vertical heterostructure design used in the current devices. The larger effective mass and the larger number of equivalent valleys and hills at *K* points give rise to a larger density of state at the *K* point compared with that at the *Λ* point and *Γ* point of *k*-space (Supplementary Note 1). Furthermore, the energy differences among these different valleys or hills (*ΔE*_Λ−ϰ_ and *ΔE*_Γ−ϰ_) are relatively small. The design of vertical heterostructure junction can create a strong vertical electric field inside the MoS_2_ flakes, which induces efficient carrier redistribution from low energy points (*Λ*, *Γ*) to the high energy points (*K*) with a larger density of state, resulting in greatly enhanced EL in the multilayer MoS_2_ flakes. The EL efficiency may be further improved by using other TMDs with smaller energy difference *ΔE*_Λ−ϰ_ and *ΔE*_Γ−ϰ_, less bulk trapping states^23^ or optimizing the device fabrication process to reduce interface impurities, defects and traps.

It should also be noted that the injected hot carriers through the tunnelling barriers in the vertical stack are also energetic enough to occupy the *K* valleys and hills and might be a possible contributing factor for the enhanced EL from the *K* point. However, without field-induced carrier redistribution in the MoS_2_, such hot carriers cannot normally sustain high-energy states (*K* valleys and *K* hills) and would quickly relax to the lowest-energy states (*Λ* point for electrons and *Γ* point for holes) before radiative recombination, because the hot-carrier relaxation or cooling time in MoS_2_ is typically on the order of sub-picosecond level while the radiative recombination time is on the order of nanosecond^40^,^41^. In addition, if the enhanced EL were a direct result of hot-carrier injection, the shape of EL spectra should strongly depend on the electric field/injection current, and the emission peaks should be very broad and not associated with distinct energy bands. In contrast, our experimental results show that the overall shape of the EL spectra in multilayer MoS_2_ remains nearly the same with the increasing electric field/injection current (Fig. 4f) and displays two distinct peaks located at the same positions as PL emission peaks. Finally, our field-induced carrier redistribution model can well explain the overall trend of EL efficiency with electric field (Fig. 4h), which cannot be consistently interpreted using the hot-carrier injection model. Together, although the injected hot carriers may be energetic enough to occupy *K* valleys and hills, such hot carriers cannot sustain high-energy states for radiative recombination, and the electric-field-induced carrier redistribution is the key factor for ensuring sufficient carrier population in *K* valleys and hills for radiative recombination and thus enhanced EL emission.

In summary, our studies for the first time report the broad-area EL emission from the entire junction area of the vertically stacked heterostructures of MoS_2_, and demonstrate unusually strong EL emission in the indirect bandgap multilayer MoS_2_. This unique EL characteristics cannot be easily achieved in other traditional indirect semiconductors (for example, Si), and is fundamentally originated from the unique electronic band structures of multilayer MoS_2_, and general for other TMD materials. Indeed, our preliminary studies indicate the electric-field-enhanced EL can also be observed in other multilayer TMDs (for example, WSe_2_, see Supplementary Note 5 and Supplementary Fig. 9). Our studies can thus not only offer a fundamental platform to probe the carrier injection, population and recombination in multilayer TMDs under high electric field, but also open up a pathway towards new types of light-emitting devices with spin- and valley polarization^25^ based on multilayer TMDs.

## Methods

### Device fabrication

MoS_2_ samples are mechanically exfoliated from a bulk MoS_2_ crystal onto a 285-nm Si/SiO_2_ substrate with alignment markers and then transferred onto the p-GaN substrate with a doping level of 2 × 10^17^ cm^−3^ grown by metalorganic chemical vapour deposition. A layer of 4-nm-thick Al_2_O_3_ was deposited on the GaN substrate using atomic layer deposition before MoS_2_ flakes were transferred. Then a layer of 60-nm-thick Al_2_O_3_ was deposited to insulate the substrate and top electrodes. Windows were defined by electron beam lithography followed by 6:1 buffered oxide etch to remove the Al_2_O_3_ on the top of MoS_2_ samples and on GaN for depositing the contact electrodes. Finally, an electron beam evaporation process was employed to deposit 5-nm Ni/50-nm Au metal thin films as top-contact electrodes at the edge of the MoS_2_ and 5-nm Pd/50-nm Au as the bottom electrode on the GaN substrate to form Ohmic contact (Supplementary Fig. 1). For vertically stacked GaN–Al_2_O_3_–MoS_2_–Al_2_O_3_-graphene devices, a 4-nm-thick Al_2_O_3_ was deposited after opening the windows. The graphene grown by the chemical vapour deposition method was transferred onto the top of the windows. Then the transferred graphene was patterned by electron-beam lithography followed by oxygen plasma etching so that the graphene only covers the MoS_2_ flakes. Finally, 20-nm Ti/50-nm Au electrodes were defined on the edge of the graphene and 5-nm Pd/50-nm Au electrodes were deposited on the GaN substrate.

### Microscopic, electrical and optical characterizations

The thickness of the MoS_2_ flakes was determined by tapping-mode atomic force microscopy (Vecco 5,000 system). The cross-section image of the vertical devices was acquired in an FEI Titan high-resolution transmission microscopy. The electrical measurement was carried out in a probe station (Lakeshore, TTP4) coupled with a precision source/measurement unit (Agilent B2902A). The PL measurement was conducted under a confocal mirco-Raman system (Horiba LABHR) equipped with a 600-mm^−1^ grating in a backscattering configuration excited by an Ar ion laser (514 nm) with an excitation power of 500 μW. The PL measurement of the GaN substrate was excited by a 257-nm laser with a power of 200 μW.

### EL measurement

The EL measurement was carried out under a home-built micro-PL system (Acton 2300i spectrometer equipped with a 150-mm^−1^ grating) combining with a precision source/measurement unit (Agilent B2902A) to force voltage or current. The EL signal was collected using a × 50 objective (numerical aperture=0.5) and acquired by liquid nitrogen-cooled CCD (Princeton instruments PyLoN 400F).

## Additional information

**How to cite this article:** Li, D. *et al.* Electric-field-induced strong enhancement of electroluminescence in multilayer molybdenum disulfide. *Nat. Commun.* 6:7509 doi: 10.1038/ncomms8509 (2015).

## Supplementary Material

Supplementary Information Supplementary Figures 1-9, Supplementary table 1, Supplementary notes 1-5 and Supplementary References.
