# Supplementary material for: Electric-field-induced strong enhancement of electroluminescence in multilayer molybdenum disulfide
Source: Nat Commun. 2015 Jul 1;6:7509. doi: 10.1038/ncomms8509 (PMC4507000; doi:10.1038/ncomms8509)
Supplement: Supplementary Information — Supplementary Figures 1-9, Supplementary table 1, Supplementary notes 1-5 and Supplementary References. [file ncomms8509-s1.pdf]

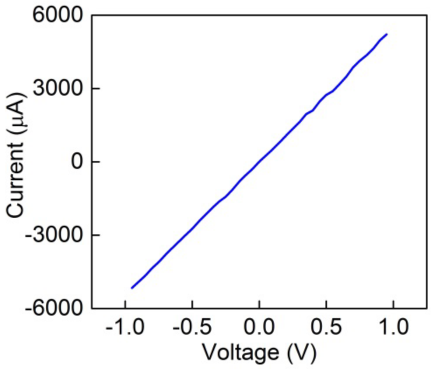


**Supplementary figure 1|** The IV curve for p-GaN with Pd/Au contacts at room temperature. The channel length is around 1 mm.

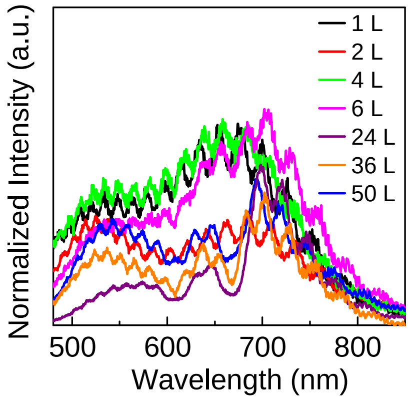


**Supplementary figure 2|** The original normalized EL spectra of various thickness $MoS_2$ flakes for GaN-$Al_2O_3$-$MoS_2$ heterostructures without subtracting the emission from GaN.

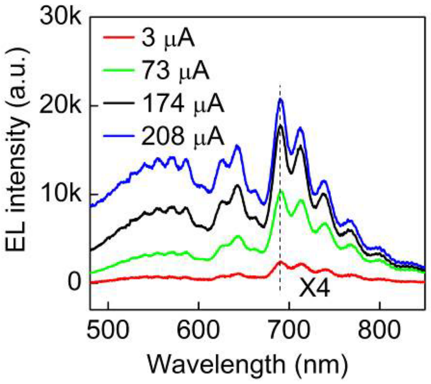


**Supplementary figure 3|** Original injection current dependent EL spectra of a 92 nm thick $MoS_2$ device with a GaN-$Al_2O_3$-$MoS_2$-$Al_2O_3$-graphene heterostructure without subtracting the emission from GaN.

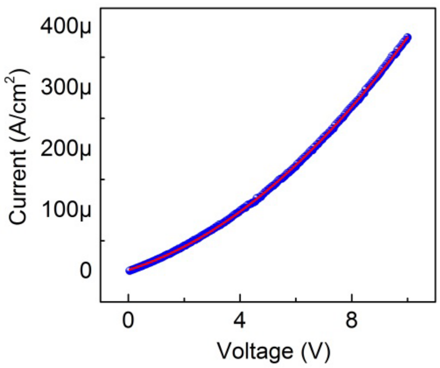


**Supplementary figure 4|** J-V characteristics of a $MoS_2$ vertical device with electrodes located at the bottom and top surfaces. The thickness of the $MoS_2$ is around 18 μm and area of the device is around 1 $cm^2$. The blue dots are experimental data and red solid curve is the fitting result.

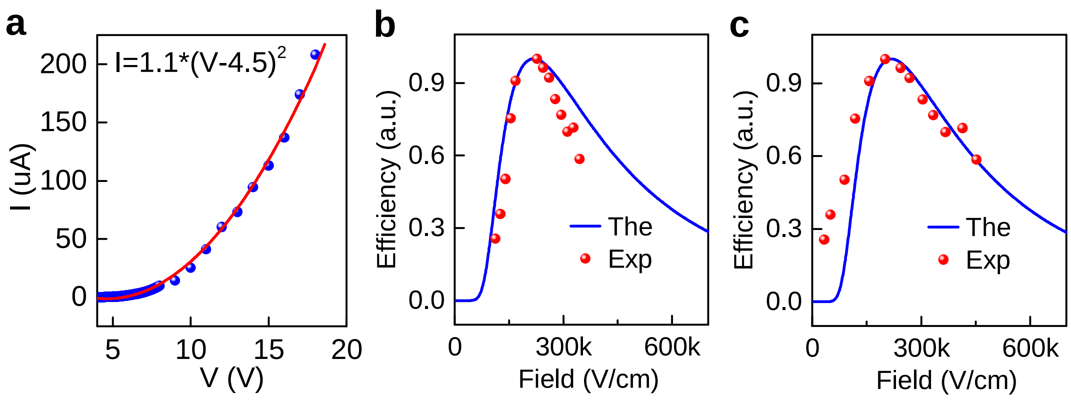


**Supplementary figure 5|** (a) IV curve for the forward bias. The discrete points are experimental result and the red solid line is the fitting result based on space charge limited current model. (b) The EL efficiency versus applied electric field for a 92 nm $MoS_2$ device. The electrical field is determined by dielectric model. (c) The EL efficiency versus applied electric field for the same 92 nm $MoS_2$ device, in which the electric field is calculated based on space charge limited current model. This model shows a better fitting with experimental results at high injection current regime.

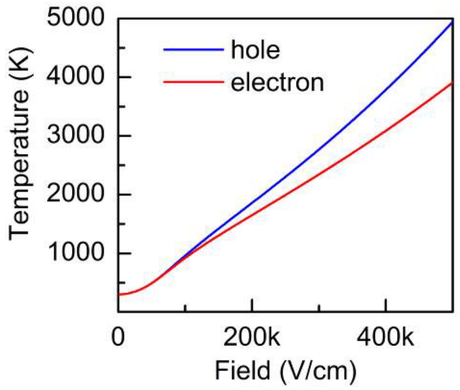


**Supplementary figure 6|** The calculated electron and hole temperature as a function of the applied electric field for bulk $MoS_2$ flakes.

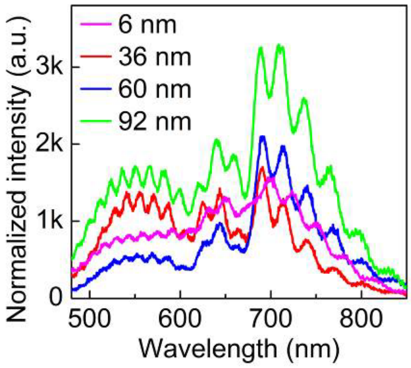


**Supplementary figure 7|** Original normalized EL spectra of various thickness $MoS_2$ flakes for GaN-$Al_2O_3$-$MoS_2$-$Al_2O_3$-graphene heterostructures without subtracting the emission from GaN.

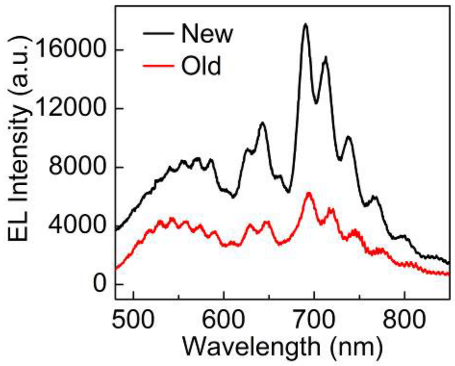


**Supplementary figure 8|** The EL spectra of the GaN-$Al_2O_3$-$MoS_2$ (old) and GaN-$Al_2O_3$-$MoS_2$-$Al_2O_3$-graphene (new) vertical heterostructures under an injection current of 240 μA for two $MoS_2$ flakes with a layer number of ~ 50.

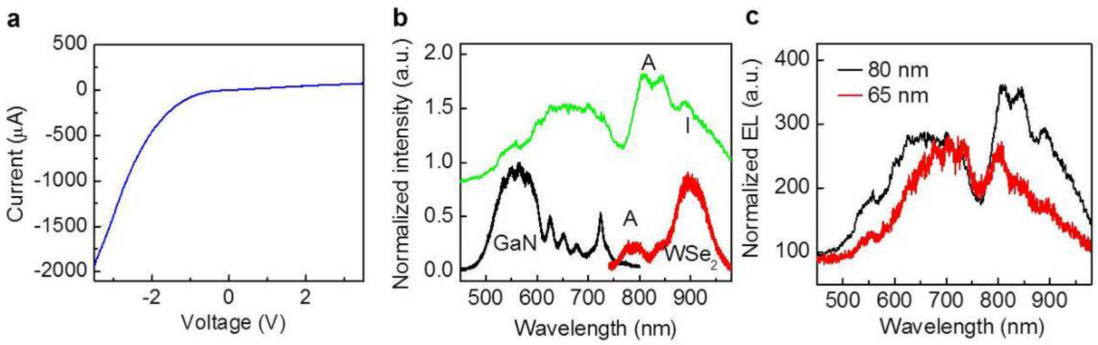


**Supplementary figure 9| EL from GaN-$Al_2O_3$-$WSe_2$-$Al_2O_3$-graphene vertical heterostructures. a,** Current versus bias voltage characteristic of the GaN-$Al_2O_3$-$MoS_2$-$Al_2O_3$-graphene vertical device. **b,** The EL spectrum of a multi-layer $WSe_2$ device under an injection current of 700 μA (upper) along with the PL spectra of the $WSe_2$ flake and GaN substrate (lower spectra). **c,** Normalized EL spectra from 65 nm and 80 nm $WSe_2$ flakes.

| | | | | | | |
|---|---|---|---|---|---|---|
| $\mu_{e\Lambda}$ | $\mu_{h\Gamma}$ | $\Delta E_{\Lambda\text{-K}}$ (bulk) | $\Delta E_{\Gamma\text{-K}}$ (bulk) | $M_{eK}$ | $M_{e\Lambda}$ | $M_{hK}$ |
| ~100 $cm^2V^{-1}\ s^{-1}$ | ~100 $cm^2V^{-1}\ s^{-1}$ | 0.3 eV | 0.3 eV | 6 | 6 | 6 |
| $M_{h\Gamma}$ | $m_{eK}$ | $m_{e\Lambda}$ | $m_{hK}$ | $m_{h\Gamma}$ | $R_e$ | $R_h$ |
| 1 | 2.70 | 0.62 | 0.70 | 0.67 | 10.6 | 6.39 |

**Supplementary table 1|** The parameters used in the calculation[1-4]. For the thin layer (4-6L), the energy difference values can be found elsewhere[5].

**Supplementary note 1:**

**Electron, hole temperature and EL efficiency**

By applying an electric field, the electrons and holes are accelerated and the kinetic energy of the carriers is increased. As a result, the carriers will redistribute among different energy valleys following the Boltzmann distribution. For example, electrons will transfer from *Λ* valley to *K* valley while holes transfer from *Γ* hill to *K* hill in multi-layer $MoS_2$. Due to the large effective mass at *K* point, the electron mobility at *K* valley is small compared with that at *Λ* valley and hole mobility at *K* is smaller than that at *Γ* valley[4]. Then the electron temperature $T_e$ and hole temperature $T_h$ can be obtained using the expressions[6]

$$T_e = T + \frac{2q\tau_e \mu_{e\Lambda}}{3k}\xi^2 (1 + \mathrm{R}_e * \exp(-\frac{\Delta E_c}{kT_e}))^{-1} \tag{1}$$

$$T_h = T + \frac{2q\tau_h \mu_{h\Gamma}}{3k}\xi^2 (1 + \mathrm{R}_h * \exp(-\frac{\Delta E_h}{kT_h}))^{-1} \tag{2}$$

where *T* is the lattice temperature, *q* is the elementary charge, *k* is Boltzmann constant, $\xi$ is electric field, $\tau_e$ and $\tau_h$ are energy relaxation time for electrons and holes respectively which are around $10^{-11}$ s [7], $\mu_{e\Lambda}$ and $\mu_{h\Gamma}$ are electron mobility at *Λ* valley and hole mobility at *Γ* hill respectively, $\Delta E_{\Lambda\text{-K}}$ is conduction band energy difference between *Λ* valley and *K* valley while $\Delta E_{\Gamma\text{-K}}$ is the valence band energy difference between *Γ* hill and *K* hill, $\mathrm{R}_e$ and $\mathrm{R}_h$ are the density-of-state (DOS) ratio for electrons between *Λ* valley and *K* valley and for holes between *Γ* hill and *K* hill, respectively. The DOS ratio for electrons and holes can be obtained by[6]

$$R_e = \frac{M_{eK}}{M_{e\Lambda}}(\frac{m_{eK}}{m_{e\Lambda}})^{3/2} \tag{3}$$

$$R_h = \frac{M_{hK}}{M_{h\Gamma}}(\frac{m_{hK}}{m_{h\Gamma}})^{3/2} \tag{4}$$

where $M_{eK}$ , $M_{e\Lambda}$ , $M_{hK}$ and $M_{h\Gamma}$ are the numbers of equivalent valleys for electrons at *K* valley and *Λ* valley and for holes at *K* hill and *Γ* hill. $m_{eK}$ , $m_{e\Lambda}$ , $m_{hK}$ and $m_{h\Gamma}$ are the effective mass for electrons at *K* valley and *Λ* valley and for holes at *K* hill and *Γ* hill.

Once we get $R_e$, $R_h$, $T_e$ and $T_h$, we can calculate the electron population ratio between *K* valley and *Λ* valley and hole population ratio between *K* hill and *Γ* hill based on[6]

$$\frac{n_2}{n_1} = \mathrm{R}_e * \exp(-\frac{\Delta E_{\Lambda\text{-K}}}{kT_e}) \tag{5}$$

$$\frac{p_2}{p_1} = \mathrm{R}_h * \exp(-\frac{\Delta E_{\Gamma\text{-K}}}{kT_h}) \tag{6}$$

Furthermore, we can obtain valley population fraction for electrons ($n_2/(n_1+n_2)$) and for holes $p_2/(p_1+p_2)$, as shown in Figure 3 (c) and (d) in the main text. The parameters used are listed in Supplementary Table 1. It should be noted that the injected carriers through tunnelling barriers are hot carriers and can populate high energy states. However, such hot carriers can not normally sustain the high energy states because the hot carrier relaxation time in $MoS_2$ is typically on the order of sub-picosecond level, much shorter than the radiative recombination time scale (on the order of nanosecond). Therefore the field-induced carrier redistribution is the primary factor responsible for K-valley (hill) population.

Supplementary Figure 6 displays the calculated electron and hole temperature. As the applied electric field increases, the electron and hole temperature increases monotonously, which gives rise to the redistribution of electrons and holes among different energy valleys. Finally, the EL efficiency η can be derived from the electron population fraction $n_2/(n_1+n_2)$, hole population fraction $p_2/(p_1+p_2)$ and radiative recombination rate *B (T)* as

$$\eta = B(T) n_2 p_2 / (I_{inj} / 2q) = B(T) * (\frac{I_{inj}}{2q}) * n_2 p_2 / ((n_1 + n_2) * (p_1 + p_2)) \tag{7}$$

where *T* is the temperature and $I_{inj}$ is the injection current. Here we assume the injected electron equals to the hole density, that is $n_1+n_2= p_1+p_2= I_{inj}/2q$. Since the thickness of the $MoS_2$ flake is thick enough to be considered as bulk, the radiative recombination rate can be expressed as a function of the temperature as[8]

$$B(T) = B(300)(\frac{300}{T})^{3/2} \tag{8}$$

Here *B(300)* is the radiative recombination rate at 300 K and we take it as a constant for all flakes we investigated in our calculations. Under the thermal equilibrium condition, the electron and hole temperature is the same as environment temperature. Here under the applied electric field, the electron and hole temperature is greatly increased and much greater than the temperature of the environment. Therefore, we used the average temperature of electron and hole temperature in the calculation. Based on Supplementary Equation (7), we obtained the electric field dependent EL efficiency (Fig. 4(h) in the main text).

**Supplementary note 2:**

**The evaluation of the out-plane mobility of $MoS_2$**

To evaluate the out-plane mobility of $MoS_2$, we have fabricated the vertical devices with electrodes located on the top and bottom surfaces of $MoS_2$ plates. Under the condition that the contacts can supply adequate carriers, the current density follows Ohm's law at the low applied voltage or low injected current density. With increasing injection current density, the current density would be mainly governed by Mott-Gurney law (space charge limited current) when the injected carrier density exceeds the intrinsic carrier density[9-12]. The total current density $J$ can expressed as[13,14]

$$J = \frac{nq\mu}{d} V + \frac{9}{8} \frac{\varepsilon\varepsilon_0\mu}{d^3} V^2 \tag{9}$$

where $n$ is carrier density, $d$ is the thickness of device, $q$ is the elementary charge, $\varepsilon$ is dielectric constant (7.6 for $MoS_2$)[15,16], $\varepsilon_0$ is vacuum dielectric constant, $\mu$ is the mobility and $V$ is the applied voltage. By applied Supplementary Equation (9) to J-V characteristics, the mobility can be evaluated.

We have fabricated $MoS_2$ vertical devices with gallium-indium eutectic contacts located on the top and bottom of the $MoS_2$ plates. Supplementary Figure 4 shows J-V characteristics of a $MoS_2$ vertical device with a thickness of 18 μm and area of ~ 1 $cm^2$. By applying Supplementary Equation (9) to fit the J-V curve, we estimated the out-plane electron mobility of $MoS_2$ to be $2.02 \times 10^{-2}$ $cm^2/Vs$, which is three orders of magnitude smaller than the in-plane mobility[17], consistent with the reported conductivity measurement[18-21].

**Supplementary note 3:**

**The evaluation of the applied electric field**

The electric field inside $MoS_2$ flakes are calculated based on the applied bias voltage and thickness of $MoS_2$ and $Al_2O_3$. We assume that the average electric field is uniformly distributed in the $MoS_2$ and $Al_2O_3$ respectively because of the presence of the insulating layer and the moderately doped $MoS_2$ and GaN, which would lead to the weak depletion electric field. At the interface between $MoS_2$ and $Al_2O_3$, the electric displacement should retain the same along the direction perpendicular to the interface, that is

$$\varepsilon_{Al_2O_3} E_{Al_2O_3} = \varepsilon_{MoS_2} E_{MoS_2} \quad (10)$$

$$\varepsilon_{Al_2O_3} E_{Al_2O_3} = \varepsilon_{GaN} E_{GaN} \quad (11)$$

Where $\varepsilon_{MoS_2}$ , $\varepsilon_{Al_2O_3}$ and $\varepsilon_{GaN}$ are dielectric constant for $MoS_2$, $Al_2O_3$ and GaN and taken as 7.6, 6 and 8.9, respectively[22,23]. $E_{MoS_2}$ , $E_{Al_2O_3}$ and $E_{GaN}$ are electric field along the direction perpendicular to the interface in $MoS_2$, $Al_2O_3$ and GaN, respectively. In our structures, almost all of the applied bias voltage drops to the junction. Thereby, the applied bias voltage $V$ can be expressed as

$$V = d_{Al_2O_3} E_{Al_2O_3} + d_{MoS_2} E_{MoS_2} + d_{GaN} E_{GaN} + V_{drop} \quad (12)$$

where $d_{MoS_2}$ and $d_{Al_2O_3}$ are the thickness of $MoS_2$ and $Al_2O_3$, respectively. $d_{GaN}$ is the width of the depletion layer in GaN, which can be obtained by $d_{MoS_2} N_{MoS_2} = d_{GaN} N_{GaN}$ ( $N_{MoS_2}$ and $N_{GaN}$ are the carrier density in $MoS_2$ and GaN respectively. We take $N_{MoS_2} = 10^{16}$ $cm^{-3}$ and $N_{GaN} = 2\times10^{17}$ $cm^{-3}$ in our calculations)[24]. $V_{drop}$ is the potential drop on the contacts, substrate, graphene and the interfaces because of the presence of the large amount of interface charge which may contribute to the potential drop[25]. With the presence of the interface charge, the Supplementary Equation (10-11) is no longer satisfied. By comparing of experimental results with theoretical ones (Supplementary Fig. 8b), the potential drop in $MoS_2$ is around 30% for the 90 nm $MoS_2$ flake device.

The dielectric model is reasonable for field calculation at low-injection current

regime, and but can not precisely evaluate the voltage distribution and electrical field in $MoS_2$ at large injected current regime, although it has previously been used to calculate the electric field in the graphene-BN-graphene tunnelling transistors[26]. To take the influence of the injected current into account, we have further developed a new model to calculate the electric field based on the current density and carrier mobility in the space charge limited current region. Since the injected carrier density surpasses the intrinsic carrier density in $MoS_2$, the current density is space-charge limited at the high injected current regime[9-12], as evidenced by the quadratic dependence of current intensity on the applied voltage (Supplementary Fig. 5a). Under such case, the average electric field $E_{avg}$ in $MoS_2$ can be calculated using[10]:

$$E_{\mathrm{avg}} = \sqrt{\frac{2J}{\mu\varepsilon\varepsilon_o}d} \tag{13}$$

where $J$ is the current density, $d$ the thickness of $MoS_2$ flakes, $\varepsilon\varepsilon_o$ the dielectric constant of $MoS_2$ flakes and $\mu$ electron mobility in $MoS_2$ along the vertical direction. The electron mobility $\mu$ in the vertical direction was evaluated to be $2.02 \times 10^{-2}$ $cm^2/Vs$ based on the space charge limited current of $MoS_2$ bulk devices.

With the new model to derive the electrical field, we found the experimental derived results agree quite well with the theoretical prediction (Supplementary Fig. 5c) except in the low injected current regime, where the injected carrier density is probably too small to reach the space-charge limited current region. In this case, the current is partly limited by tunnelling through the $Al_2O_3$ dielectric layer, leading to an underestimation of the electrical field in $MoS_2$. At lower injection current limit, the dielectric model should give a better estimation of the electrical field (Supplementary Fig. 5b).

**Supplementary note 4:**

**EL extraction efficiency and effective thickness**

The EL from inside of the samples has to propagate to the top surface to be detected. During the propagation process, part of the emitted light from inside of the sample would be reabsorbed by the sample itself and converted to heat due to the low radiative recombination rate in the indirect bandgap semiconductors. For simplicity, we consider each layer as a light emitter and take the optical density of each layer as $A$ and emission intensity of each layer as $I_0$. For an $N$-layer $MoS_2$ flake, the detected intensity emitted from the bottom layer can be expressed as

$$I = I_0 \exp(-(N-1)A) \tag{14}$$

Therefore, the total intensity detected is

$$\begin{aligned} I_{tot} &= I_0 + I_0 \exp(-A) + I_0 \exp(-2A) + \cdots + I_0 \exp(-(N-1)A) \\ &= I_0 * (1 + \exp(-A) \frac{1-\exp(-(N-1)A)}{1-\exp(-A)}) \end{aligned} \tag{15}$$

The EL extraction efficiency can be expressed as

$$\eta_e = \frac{I_{tot}}{NI_0} = (1 + \exp(-A) \frac{1-\exp(-(N-1)A)}{1-\exp(-A)}) / N \tag{16}$$

And the effective thickness can be defined as

$$N_{eff} = N * \eta_e \tag{17}$$

We have calculated effective layer number and EL extraction efficiency as a function of the actual layer number. The absorption coefficient was taken as 11.5 $\mu m^{-1}$ and the thickness of each layer is 0.65 nm. The effective layer number increases first and reaches a stable value around 120 nm even if the actual layer number is much larger. While the EL extraction efficiency monotonously decreases. In the viewpoint of the EL extraction efficiency, very thick samples cannot be effectively used for lighting. On the other hand, the percentage of injected carrier leakage will be smaller in the thick $MoS_2$ flakes, which leads to a stronger emission intensity. Here in our vertical structures, part of the injected carriers leak into the electrodes and don't contribute to photon emission[27].

Thus, taking those two factors into consideration and assuming each layer has the same emission intensity, the integrated emission intensity $I_{int}$ can be expressed as

$$I_{\text{int}} = \eta_{\text{e}} N B(T) n_2 p_2 = N_{eff} B(T) n_2 p_2 \tag{18}$$

Based on Supplementary Equation (18), we obtained the calculation results in Figure 5b in the main text.

## Supplementary note 5:

## EL from GaN-$Al_2O_3$-$WSe_2$-$Al_2O_3$-graphene heterostructures

We have also created GaN-$Al_2O_3$-$WSe_2$-$Al_2O_3$-Graphene heterostructures with various thickness $WSe_2$ flakes, in which an n-type GaN was used to inject electrons into p-type $WSe_2$ flakes. The output characteristic of the vertically stacked heterostructures shows the expected rectification behaviour (Supplementary Fig. 9a). The EL spectrum of an 80-layer $WSe_2$ device is shown in Supplementary Figure 9b, along with the PL spectra of n-GaN and $WSe_2$. Close comparison of EL spectrum and PL spectra, we can assign the EL peaks at 808 nm to the exciton A and at 890 nm to indirect bandgap emission of multi-layer $WSe_2$ [28,29] by taking account into the redshift of the EL emission peaks due to the self-heating effect[30]. The emission peak located around 600 nm can be attributed to the defect emission of Si-doped n-GaN[31]. Similar to vertically stacked heterostructures with $MoS_2$, stronger EL was also observed in the thicker $WSe_2$ flake than thinner one (Supplementary Fig. 9c).

**Supplementary References**


1 Bao, W. *et al.* High mobility ambipolar $MoS_2$ field-effect transistors: Substrate and dielectric effects. *Appl. Phys. Lett.* **102**, 012111 (2013).

2 Splendiani, A. *et al.* Emerging photoluminescence in Monolayer $MoS_2$. *Nano Lett.* **10**, 1271-1275 (2010).

3 Peelaers, H. & Van de Walle, C. G. Effects of strain on band structure and effective masses in $MoS_2$. *Phys. Rev. B* **86**, 241401 (2012).

4 Jin, W. *et al.* Direct measurement of the thickness-dependent electronic band structure of $MoS_2$ using angle-resolved photoemission spectroscopy. *Phys. Rev. Lett.* **111**, 106801 (2013).

5 Mak, K. F. *et al.* Atomically thin $MoS_2$: A new direct-gap semiconductor. *Phys. Rev. Lett.* **105**, 136805 (2010).

6 Sze, S. M. & Ng, K. K. *Physics of semiconductor devices*. (John Wiley & Sons, 2006).

7 Kumar, N. *et al.* Charge carrier dynamics in bulk $MoS_2$ crystal studied by transient absorption microscopy. *J. Appl. Phys.* **113**, 133702 (2013).

8 Zhang, J., Li, D., Chen, R. & Xiong, Q. Laser cooling of a semiconductor by 40 kelvin. *Nature* **493**, 504-508 (2013).

9 Rose, A. Space-charge-limited currents in solids. *Phys. Rev.* **97**, 1538-1544 (1955).

10 Dacey, G. Space-charge limited hole current in germanium. *Phys. Rev.* **90**, 759-763 (1953).

11 Shockley, W. & Prim, R. Space-charge limited emission in semiconductors. *Phys. Rev.* **90**, 753-758 (1953).

12 Lampert, M. A. Simplified theory of space-charge-limited currents in an insulator with traps. *Phys. Rev.* **103**, 1648-1656 (1956).

13 Ma, L. *et al.* Epitaxial growth of large area single-crystalline few-layer $MoS_2$ with high space charge mobility of 192 $cm^2$ $V^{-1}$ $s^{-1}$. *Appl. Phys. Lett.* **105**, 072105 (2014).

14 Ghatak, S. & Ghosh, A. Observation of trap-assisted space charge limited conductivity in short channel $MoS_2$ transistor. *Appl. Phys. Lett.* **103**, 122103 (2013).

15 Kim, S. *et al.* High-mobility and low-power thin-film transistors based on multilayer $MoS_2$ crystals. *Nature Commun.* **3**, 1011 (2012).

16 Frindt, R. & Yoffe, A. Physical properties of layer structures: optical properties and photoconductivity of thin crystals of molybdenum disulphide. *Proc. R. Soc. A* **273**, 69-83 (1963).

17 Radisavljevic, B. *et al.* Single-layer $MoS_2$ transistors. *Nature Nanotech.* **6**, 147-150 (2011).

18 Thakurta, S. R. G. & Dutta, A. K. Electrical conductivity, thermoelectric power and hall effect in p-type molybdenite ($MoS_2$) crystal. *J. Phys. Chem. Solids.* **44**, 407-416 (1983).

19 Wieting, T. J. Electrical conductivity of thin single crystals of the IVB-VIB dichalcogenides. *J. Phys. Chem. Solids.* **31**, 2148-2151 (1970).

20 Evans, B. & Young, P. Optical absorption and dispersion in molybdenum disulphide. *Proc. R. Soc. A* **284**, 402-422 (1965).

21 Dutta, A. K. Electrical conductivity of molybdenite crystals. *Nature* **159**, 447 (1947).

22 Levinshtein, M. E., Rumyantsev, S. L. & Shur, M. S. *Properties of Advanced Semiconductor Materials: GaN, AIN, InN, BN, SiC, SiGe.* (John Wiley & Sons, 2001).

23 Kim, S. *et al.* Realization of a high mobility dual-gated graphene field-effect transistor with $Al_2O_3$ dielectric. *Appl. Phys. Lett.* **94**, 062107 (2009).

24 Grant, A. J., Griffiths, T. M., Pitt, G. D. & Yoffe, A. D. The electrical properties and the magnitude of the indirect gap in the semiconducting transition metal dichalcogenide layer crystals. *J. Phys. C. Solid State Phys.* **8**, L17 (1975).

25 Esposto, M. *et al.* Electrical properties of atomic layer deposited aluminum oxide on gallium nitride. *Appl. Phys. Lett.* **99**, 133503 (2011).

26 Britnell, L. *et al.* Field-effect tunneling transistor based on vertical graphene heterostructures. *Science* **335**, 947-950 (2012).

27 Yamakoshi, S. *et al.* Direct observation of electron leakage in InGaAsP/InP double heterostructure. *Appl. Phys. Lett.* **40**, 144-146 (1982).

28 Tonndorf, P. *et al.* Photoluminescence emission and Raman response of monolayer $MoS_2$, $MoSe_2$, and $WSe_2$. *Opt. Express* **21**, 4908-4916 (2013).

29 Zhao, W. *et al.* Evolution of Electronic Structure in Atomically Thin Sheets of $WS_2$ and $WSe_2$. *ACS Nano* **7**, 791-797 (2012).

30 Gong, Z. *et al.* Size-dependent light output, spectral shift, and self-heating of 400 nm InGaN light-emitting diodes. *J. Appl. Phys.* **107**, 013103 (2010).

31 Lee, I. *et al.* Band-gap narrowing and potential fluctuation in Si-doped GaN. *Appl. Phys. Lett.* **74**, 102-104 (1999).
